# Announcing the 2018 *Toxins* Travel Awards for Post-Doctoral Fellows

**DOI:** 10.3390/toxins10010046

**Published:** 2018-01-19

**Authors:** Jay W. Fox

**Affiliations:** Department of Microbiology, University of Virginia, Charlottesville, VA 22908-0734, USA; jwf8x@virginia.edu

This year we enjoyed a large number of very highy meritorious applications for our annual Toxin Travel Awards. It was not an easy task to select the top two candidates. Nevertheless, with the assistance of our Section Editors, we have identified two outstanding candidates. Thus, as Editor-in-Chief of Toxins, I am pleased to announce the winners of the Toxins Travel Awards for 2018:

Travel awards were granted to Dr. Kwok-ho Lam, a postdoctoral fellow in Dr. Rongsheng Jin’s lab at University of California Irvine, USA, and to Dr. Anneleen Pletinck, a postdoctoral fellow in Prof. Griet Glorieux’s lab at Campus Ghent University Hospital, Belgium (Figure 1).

Dr. Kwok-ho Lam received his Ph.D in Molecular Biotechnology from the Chinese University of Hong Kong in 2011, where he worked in the laboratory of Prof. Shannon Wing-ngor Au. He then joined the laboratory of Prof. Rongsheng Jin at the University of California Irvine as a Postdoc. His research mainly focuses on understanding the structure and function of botulinum neurotoxins (BoNTs) produced by *Clostridium botulinum*, including how BoNTs transform from a soluble protein to a membrane-embedded protein-conducting channel, how BoNTs specifically recognize host receptors, and structure-based development of novel inhibitors and antitoxins against BoNTs. Dr. Lam has co-authored 18 peer-reviewed research publications, including papers published in *Nature* and *Nature Structural & Molecular Biology*. He will use the *Toxins* Travel Award to attend the Gordon Research Conference on “Bacterial Pathogenesis: From Pathogen Physiology to Interactions with Host Microbiota and Immune System” in Waterville Valley, NH, USA, 8–13 July 2018.

Dr. Anneleen Pletinck received her Ph.D from Ghent University, Belgium in 2012, where she worked in the laboratory of Prof. Raymond Vanholder, one of the founders of the EUTox consortium. Her thesis involved a detailed study of the peritoneal membrane as a window for microvascular pathophysiology in chronic kidney disease, focusing on uremic toxins. Anneleen is currently working as a postdoctoral researcher in the laboratory of Prof. Wim Van Biesen and Prof. Griet Glorieux and continues to pursue her interest in the effect of uremic toxins on the endothelial glycocalyx, playing an important role in the increased risk for cardiovascular disease, the major cause of death in patients with chronic kidney disease. Dr. Pletinck has co-authored 16 peer-reviewed papers. She has published as a first author in high impact journals as Nature Reviews Nephrology and Journal of the American Society of Nephrology. Dr. Pletinck will attend the annual research meeting of the European Uremic Toxin work group in Utrecht, The Netherlands, 16–17 March 2018.

The editors, managing editor and editorial board members join me in congratulating Dr. Kwok-ho Lam and Dr. Anneleen Pletinck on winning 2018 *Toxins* Travel Awards. Toxins is proud to support these young scientists working in the field of toxinology and wish them further success in their careers. We are grateful to all who submitted applications—thank you for letting us get to know you and your work. The future of toxinology looks very bright indeed. Finally, we are grateful to MDPI for their generous support of young scholars, helping them to share their work on the international stage.

## Figures and Tables

**Figure 1:**
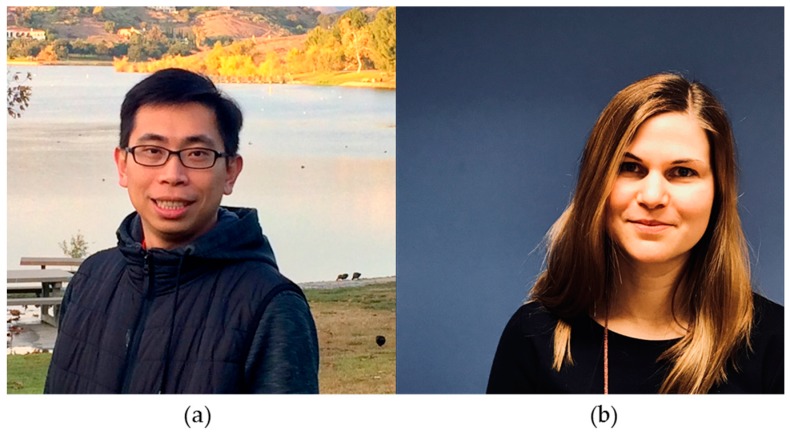
(**a**) Dr. Kwok-ho Lam; (**b**) Dr. Anneleen Pletinck.

